# Psychotropic prescribing in UK primary care during the COVID-19 pandemic: national interrupted time-series analysis

**DOI:** 10.1192/bjo.2026.12054

**Published:** 2026-07-28

**Authors:** Amanj Kurdi, Abdullah Alrahmani, Saher Bakar, Chris Johnson, Brian Godman

**Affiliations:** Strathclyde Institute of Pharmacy and Biomedical Sciences, University of Strathclyde, Glasgow, UK; College of Pharmacy, Al-Kitab University, Kirkuk, Iraq; Department of Public Health Pharmacy and Management, School of Pharmacy, Sefako Makgatho Health Sciences University, Pretoria, South Africa; College of Pharmacy, Hawler Medical University, Erbil, Iraq; Department of Engineering, College of Engineering and Technology, University of Technology and Applied Sciences, Muscat, Oman; Primary Care, NHS Greater Glasgow and Clyde, Glasgow, UK; Antibiotic Policy Group, Institute for Infection and Immunity, City St. George’s, University of London, UK

**Keywords:** Psychotropic prescribing, COVID-19, drug utilisation, mental health services, UK

## Abstract

**Background:**

The COVID-19 pandemic disrupted healthcare delivery and coincided with increased psychological distress worldwide. In the UK, restrictions on healthcare access, rapid adoption of remote consultations and pressures on mental health services may have influenced prescribing of psychotropic medications. However, evidence comparing prescribing trends across the four UK nations during the pandemic remains limited.

**Aims:**

To examine trends in community prescribing of psychotropic medicines across England, Scotland, Wales and Northern Ireland before and during the COVID-19 pandemic.

**Method:**

We conducted a repeated cross-sectional study of national Prescription Cost Analysis data-sets covering March 2019 to March 2023. Monthly utilisation of antidepressants, anxiolytics/hypnotics, antipsychotics and antidementia medicines was measured, using two indicators: number of items dispensed per 1000 inhabitants and defined daily doses per 1000 inhabitants per day. Interrupted time-series regression was applied to assess changes in prescribing levels and trends associated with the first (March 2020) and second (November 2020) UK national lockdowns.

**Results:**

Psychotropic prescribing increased across most medicine classes during the study period. Antidepressant utilisation increased by 24.6% across the UK, representing the largest rise among psychotropic medicines. Antipsychotic prescribing increased by 2.7%; anxiolytic/hypnotic prescribing showed modest overall change (2.7%), but this varied between nations, increasing in Scotland (7.3%) and Northern Ireland (7.4%), and declining in Wales (−9.5%). Antidementia medicines increased modestly overall (9.3%), with variation between countries. Interrupted time-series analyses indicated that most changes occurred gradually rather than abrupt shifts following lockdowns.

**Conclusions:**

Psychotropic prescribing increased during the COVID-19 period, but largely reflected continuation of pre-existing trends rather than immediate effects of lockdown restrictions.

The COVID-19 pandemic caused substantial disruption to health systems and population mental health worldwide, including in the UK.^1^ To limit transmission of SARS-CoV-2, the UK implemented national lockdowns in March and November 2020, alongside other public health restrictions that significantly altered access to healthcare services.^2^ During the early stages of the pandemic, primary care activity declined and services rapidly transitioned to remote consultations.^3^ Mental healthcare pathways were also affected, with reductions in referrals, service contacts and routine monitoring during early lockdown periods.^4^ Similar disruptions to mental health services were observed across Europe.^5^ At the same time, multiple studies reported increases in psychological distress in the general population. National surveys in the UK identified substantial rises in anxiety and depressive symptoms during the pandemic,^6,7^ consistent with global estimates suggesting a 25% increase in the prevalence of anxiety and depression worldwide.^8^ However, the burden was not evenly distributed, with vulnerable groups and individuals who experience socioeconomic disadvantage facing greater impacts on mental health.^9^

Psychotropic medicines, including antidepressants, anxiolytics, antipsychotics and antidementia drugs, are central to the management of mental health and neuropsychiatric conditions.^10^ Changes in their utilisation may therefore provide important insights into population mental health needs, access to care and the resilience of mental health services during periods of disruption. Evidence from several countries suggests that the pandemic was associated with heterogeneous prescribing responses. Studies from Scandinavia and Southern Europe reported increases in antidepressant and anxiolytic utilisation during the pandemic,^11–13^ whereas other work highlighted disruption to dementia diagnosis and treatment pathways.^14^ Evidence from the UK remains limited and inconsistent. Some studies reported temporary increases in antidepressant prescribing during early phases of the pandemic,^15^ whereas others found relatively stable patterns for anxiolytics and hypnotics.^15,16^ Antipsychotic prescribing also changed in certain patient groups, particularly among people with dementia.^17^ However, most existing analyses have focused on single drug classes, short follow-up periods or individual UK nations. Given the devolved structure of the UK health system and variation in policy responses across the four nations,^18^ a comprehensive national comparison is needed. This study aimed to examine trends in community prescribing of antidepressants, anxiolytics/hypnotics, antipsychotics and antidementia medicines across England, Scotland, Wales and Northern Ireland before and during the COVID-19 pandemic, by using national prescribing data-sets and interrupted time-series analysis.

## Method

### Study design and data sources

We conducted a retrospective, observational, repeated cross-sectional study to examine the impact of the COVID-19 pandemic on community utilisation of psychotropic medication across the four countries of the UK. The analysis used nationally representative Prescription Cost Analysis (PCA) data-sets for England,^19^ Scotland,^20^ Wales^21^ and Northern Ireland.^22^ These data-sets record all items prescribed in primary care and subsequently dispensed in community pharmacies, providing monthly aggregated information on drug name, strength, formulation and quantity supplied. The study period spanned March 2019 to March 2023, capturing 12 months before the first UK national lockdown in March 2020, the acute pandemic and lockdown phases, and an extended recovery period following relaxation of major COVID-19 restrictions (36 months) thereafter. This time frame encompasses both national lockdown periods introduced by the UK Government,^2^ sustained alterations to service delivery (including digital transformation of primary care) and fluctuations in prescribing capacity and healthcare access documented during the pandemic. Population denominators were derived from the Office for National Statistics mid-year estimates for each country and year.^23^ This study is reported in accordance with the Strengthening the Reporting of Observational Studies in Epidemiology statement for cross-sectional studies.

### Study medicines

The study included all psychotropic medications falling within four therapeutic categories: hypnotics/anxiolytics, antidepressants, antipsychotics and antidementia drugs (Supplementary File 1). Medicines were selected according to British National Formulary classifications^24^ and consistent with prior research examining psychotropic medications use during the pandemic.^11,14,15^ All formulations and strengths of medicines licenced within these classes and dispensed in community settings were included. Hospital-only medicines and private prescriptions were not captured by PCA data and were therefore excluded. Antidementia medicines included acetylcholinesterase inhibitors and memantine; antipsychotics included typical and atypical agents; antidepressants covered selective serotonin reuptake inhibitors, serotonin norepinephrine reuptake inhibitors, tricyclic antidepressants and other classes; and anxiolytics included benzodiazepines and related agents.

### Study outcomes

The primary outcomes were the monthly utilisation trends for each class of central nervous system medicines, measured with two standardised pharmacoepidemiological indicators: the number of dispensed items per 1000 inhabitants (NTI) and the defined daily dose per 1000 inhabitants per day (DID). NTI was calculated by extracting the monthly number of dispensed items from the PCA data-set for each country, dividing this value by the corresponding mid-year population estimate and multiplying the result by 1000 to generate the number of items dispensed per 1000 inhabitants. DID was calculated by summing the total amount of each medicine dispensed in milligrams each month, derived by multiplying the strength in milligrams by the quantity dispensed. This total was divided by the World Health Organization-defined defined daily dose for that medicine,^25^ to obtain the number dispensed. The resulting value was then divided by the mid-year population estimate, multiplied by 1000 and standardised by the number of days in the month to produce. DID accounts for differences in dosage strength, treatment duration and population size across regions. These outcomes were chosen to ensure consistency with established pharmacoepidemiological research and to allow alignment with international evaluations of psychotropic utilisation during the COVID-19 pandemic.^11,15^

### Data analysis

To characterise changes in psychotropic medication utilisation before and during the COVID-19 pandemic, we applied descriptive statistical methods to calculate absolute and relative differences in NTI and DID between March 2019 and March 2023. To formally evaluate the impact of the first and second national lockdowns and associated public health measures, segmented regression with an interrupted time-series design was undertaken.^26^ This approach is recognised as a robust method for assessing temporal changes associated with natural experiments, and has been widely applied in recent studies examining the effects of the pandemic on psychotropic prescribing.^11,12^ The regression model estimated baseline pre-pandemic utilisation trends, immediate changes in level following the first (March 2020) and second (November 2020) UK national lockdowns, and changes in slope during the periods after these interventions. Monthly time points across the entire study period were incorporated to allow detection of both abrupt and sustained shifts in utilisation patterns. Diagnostic checks were performed to assess for autocorrelation and seasonal variation in model residuals, ensuring validity of the statistical estimates. All analyses were conducted with Stata Statistical Software, Release 15 (StataCorp LLC, College Station, Texas, USA; https://www.stata.com/stata15/) running on Microsoft Windows, and consistent with methodological approaches used in PCA-based drug utilisation studies, and findings were interpreted in the context of the broader evidence regarding mental health burden, service disruption and prescribing behaviour during the pandemic.^11,14,15^

## Results

### Monthly NTIs

**Fig. 1:**
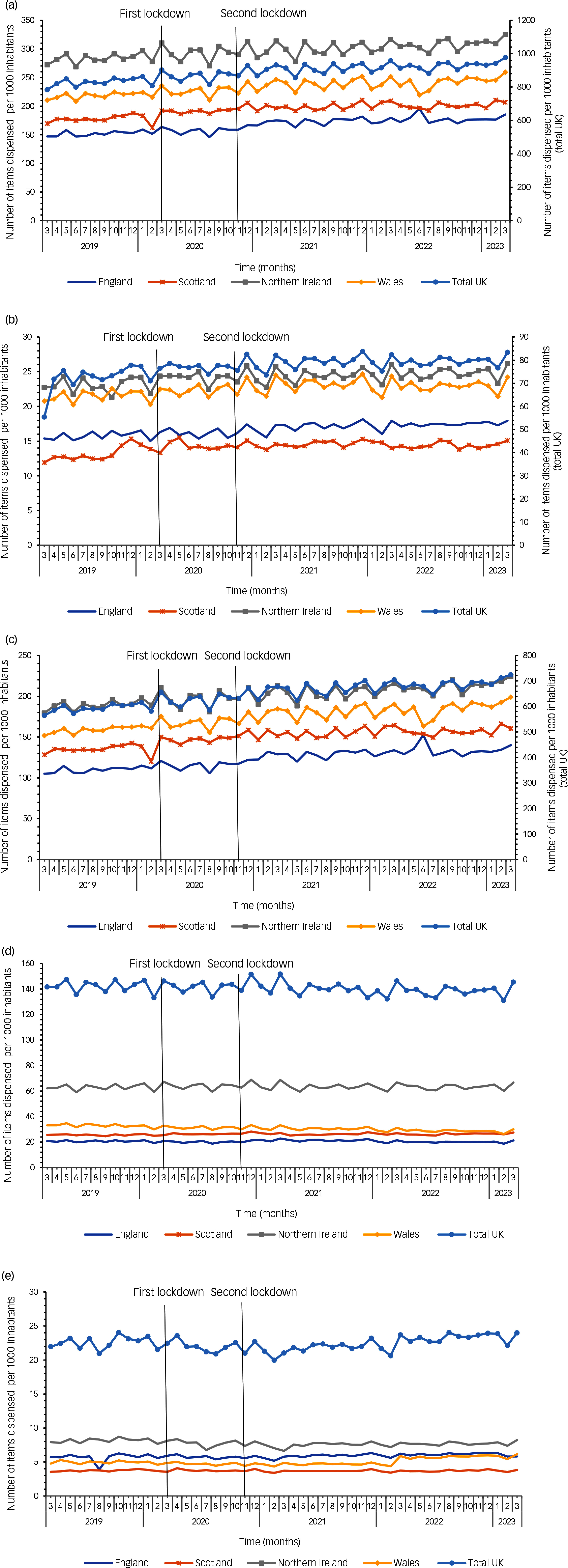
Fig. 1 long description. Prescribing pattern of psychotropic medications expressed as total number of items dispensed per 1000 inhabitants for the UK and each UK country from March 2019 to March 2023. (a) All psychotropic medications, (b) antipsychotics, (c) antidepressants, (d) anxiolytics/hypnotics and (e) antidementia drugs.

Between March 2019 and March 2023, psychotropic medication prescribing increased across the UK (Fig. 1). A total of approximately 39 108 psychotropic medicine items were dispensed during the study period, with antidepressants accounting for nearly 70% of all prescriptions (*n* = 27 338). Antidepressant utilisation increased substantially across the UK, rising by 24.6% overall. Wales showed the largest relative increase (23.3%), whereas Scotland demonstrated the smallest (20.0%) (Table 1). Segmented regression indicated limited immediate effects of national lockdowns on antidepressant prescribing, although England showed a modest reduction in the rate of increase following the second lockdown (*β*_5_ = −0.64; 95% CI −1.19 to −0.08) (Table 2). Anxiolytics and hypnotics accounted for 17.6% of dispensed items (*n* = 6892). Trends varied between nations. Scotland showed a 7.3% increase, whereas Wales experienced a 9.5% reduction over the study period. Northern Ireland consistently recorded the highest utilisation levels (Table 2; Fig. 1(c)). A significant increase in England occurred immediately after the second lockdown (*β*_4_ = 1.60; 95% CI 0.40–2.80). Antipsychotic prescribing increased modestly overall across the UK (2.7%), with significant upward trends observed in all four countries (Table 2; Fig. 1(a)). Northern Ireland had the highest utilisation throughout the study period. Scotland showed a small but significant reduction in trend following the first lockdown (*β*_3_ = −0.25; 95% CI −0.44 to −0.05). Antidementia prescribing showed minimal overall change at the UK level. However, utilisation increased in England (1.7%) and Wales (29.0%) during the study period, whereas Northern Ireland consistently reported the highest levels and Scotland reported the lowest.

**Table 1:** Absolute, relative and average monthly changes in the utilisation trends of psychotropic medications across England, Scotland, Wales and Northern Ireland over the study period March 2019 to March 2023 Table 1 long description.

Utilisation metrics	UK (total)	England	Scotland	Wales	Northern Ireland
Number of dispensed items per 1000 inhabitants					
All psychotropic medications					
Absolute change (relative % change)	193.0 (24.6)	38.2 (26.0)	37.3 (22.0)	48.9 (23.3)	53.2 (19.5)
Monthly average change	2.8*	0.7*	0.6*	0.7*	0.7*
Antipsychotics					
Absolute change (relative % change)	27.9 (50.4)	2.5 (16.5)	3.2 (26.6)	3.4 (16.6)	3.4 (15.0)
Monthly average change	0.2*	0.01*	0.03*	0.04*	0.04*
Antidepressants					
Absolute change (relative % change)	123.9 (24.6)	32.2 (31.2)	18.8 (20.0)	37.7 (23.3)	35.1 (24.2)
Monthly average change	2.6*	0.7*	0.6*	0.7*	0.6*
Anxiolytics/hypnotics					
Absolute change (relative % change)	3.8 (2.7)	0.5 (2.5)	1.9 (7.3)	−3.1 (−9.5)	4.6 (7.4)
Monthly average change	−0.1	−0.01	0.02*	−0.1*	0.01
Antidementia					
Absolute change (relative % change)	2.0 (9.3)	0.01 (1.7)	0.3 (7.5)	1.4 (29.0)	0.3 (3.7)
Monthly average change	0.02	0.01*	−0.001	0.02*	−0.01*
Number of defined daily doses per 1000 inhabitants per day					
All psychotropic medications					
Absolute change (relative % change)	175.9 (25.1)	38.4 (30.3)	36.6 (23.0)	47.2 (26.3)	53.7 (23.0)
Monthly average change	2.9*	0.7*	0.7*	0.7*	0.8*
Antipsychotics					
Absolute change (relative % change)	5.9 (18.2)	1.3 (20.0)	1.4 (19.7)	1.6 (19.1)	1.7 (15.6)
Monthly average change	0.08*	0.02*	0.02*	0.02*	0.02*
Antidepressants					
** **Absolute change (relative % change)	159.1 (28.2)	35.0 (33.3)	32.0 (24.9)	47.3 (31.1)	44.9 (25.0)
Monthly average change	2.6*	0.7*	0.6*	0.7*	0.6*
Anxiolytics/hypnotics					
Absolute change (relative % change)	10.0 (12.3)	1.5 (13.3)	3.0 (15.3)	−1.4 (−10.0)	6.9 (19.3)
Monthly average change	0.2*	0.02*	0.07*	−0.04*	0.1*
Antidementia					
Absolute change (relative % change)	0.7 (3.6)	0.6 (15.1)	0.2 (4.2)	−0.2 (−4.3)	0.2 (2.1)
Monthly average change	−0.02*	0.01*	−0.003*	−0.01*	−0.01*

*Indicates *p* < 0.05, obtained from trend/liner regression analysis.

**Table 2:** Segmented regression analysis of the monthly number of items dispensed per 1000 inhabitants for psychotropic medications across England, Scotland, Wales and Northern Ireland over the study period March 2019 to March 2023

Psychotropic medications	Baseline trend (*β*_1_)	Level change immediately after first lockdown (*β*_2_)	Time trend after first lockdown (*β*_3_)	Level change immediately after second lockdown (*β*_4_)	Time trend after second lockdown (*β*_5_)
All					
UK	3.20 (−1.20 to 7.50)	23.80 (−25.00 to 72.70)	−3.80 (−12.90 to 5.20)	25.80 (−12.80 to 64.40)	2.50 (−5.50 to 10.50)
England	0.60 (−0.40 to 1.60)	2.80 (−8.20 to 13.90)	−0.90 (−2.90 to 1.10)	**12.00 (3.30−20.70)**	0.70 (−1.10 to 2.50)
Scotland	0.40 (−0.50 to 1.30)	**10.80 (0.09−21.50)**	−0.10 (−2.10 to 1.90)	5.00 (−3.40 to 13.50)	−0.01 (−1.70 to 1.70)
Wales	0.70 (−0.70 to 2.10)	3.90 (−12.40 to 20.20)	−0.60 (−3.60 to 2.40)	5.70 (−7.20 to 18.60)	0.40 (−2.20 to 3.10)
Northern Ireland	0.90 (−0.90 to 2.60)	8.30 (−11.40 to 28.10)	−1.60 (−5.20 to 2.10)	3.70 (−11.90 to 19.00)	1.30 (−1.90 to 4.60)
Antipsychotics					
UK	**0.92 (1.46−0.38)**	0.12 (6.25 to −6.01)	−1.00 (0.14 to −2.13)	1.60 (6.44 to −3.24)	0.15 (1.15 to −0.86)
England	0.04 (0.13 to −0.04)	0.46 (1.50 to −0.50)	−0.12 (0.07 to −0.31)	0.79 (1.60 to −0.03)	2.23 (12.80 to −8.32)
Scotland	**0.22 (0.32−0.14)**	−0.09 (0.97 to −1.17)	**−0.25 (−0.05 to −0.44)**	0.29 (1.14 to −0.55)	0.02 (0.20 to −0.15)
Wales	0.04 (0.19 to −0.11)	0.36 (2.06 to −1.35)	0.04, (0.35 to −0.28)	0.35 (1.7 to −0.99)	−0.07 (0.21 to −0.35)
Northern Ireland	0.01 (0.17 to −0.15)	1.37 (3.17 to −0.43)	−0.07 (0.26 to −0.4)	0.16 (1.58 to −1.26)	0.09 (0.38 to −0.21)
Antidepressants					
UK	1.38 (−2.51 to 5.27)	3.15 (−26.67 to 32.95)	0.99 (−3.21 to 5.19)	0.45 (−26.70 to 27.61)	−1.47 (−4.04 to 1.09)
England	0.40 (−0.44 to 1.25)	−1.70 (−8.15 to 4.75)	0.50 (−0.41 to 1.41)	−1.25 (−7.12 to 4.62)	**−0.64 (−1.19 to −0.08)**
Scotland	0.41 (−0.22 to 1.03)	1.75 (−3.03 to 6.52)	−0.15 (−0.82 to 0.52)	1.28 (−3.06 to 5.63)	−0.23 (−0.64 to 0.18)
Wales	0.37 (−1.03 to 1.77)	1.24 (−9.48 to 11.96)	0.43 (−1.08 to 1.94)	0.42 (−9.34 to 10.19)	−0.72 (−1.64 to 0.21)
Northern Ireland	0.20 (−1.11 to 1.52)	1.86 (−8.24 to 11.95)	0.22 (−1.21 to 1.64)	−0.001 (−9.20 to 9.20)	0.11 (−0.76 to 0.98)
Anxiolytics/hypnotics					
UK	−0.20 (−1.00 to 0.60)	2.40 (−6.80 to 11.60)	−0.10 (−1.80 to 1.60)	1.80 (−5.40 to 9.10)	0.10 (−1.40 to 1.60)
England	−0.03 (−0.20 to 0.10)	−0.1 (−1.70 to 1.40)	−0.03 (−0.30 to 0.30)	**1.60 (0.40−2.80)**	−0.001 (−0.30 to 0.30)
Scotland	−0.02 (−0.10 to 0.10)	0.50 (−0.80 to 1.80)	0.10 (−0.20 to 0.30)	−0.04 (−1.10 to 0.90)	−0.05 (−0.30 to 0.20)
Wales	−0.2 (−0.40 to 0.03)	−0.01 (−2.30 to 2.30)	0.08 (−0.30 to 0.50)	0.400 (−1.40 to 2.20)	−0.02 (−0.40 to 0.40)
Northern Ireland	0.02 (−0.40 to 0.50)	2.10 (−2.80 to 6.90)	−0.20 (−1.10 to 0.70)	0.50 (−3.30 to 4.40)	0.20 (−0.60 to 0.90)
Antidementia					
UK	0.11 (−0.08 to 0.29)	−0.39 (−2.10 to 1.32)	−0.32 (−0.69 to 0.04)	−0.17 (−1.46 to 1.12)	0.30 (−0.02 to 0.61)
England	0.04 (−0.04 to 0.12)	0.02 (−0.75 to 0.78)	−0.08 (−0.25 to 0.08)	0.04 (−0.54 to 0.61)	0.06 (−0.08 to 0.20)
Scotland	0.03 (−0.04 to 0.06)	−0.14 (−0.41 to 0.14)	−0.03 (−0.08 to 0.03)	−0.09 (−0.29 to 0.12)	0.00 (−0.05 to 0.05)
Wales	−0.01 (−0.07 to 0.06)	−0.12 (−0.74 to 0.51)	−0.03 (−0.16 to 0.10)	−0.35 (−0.82 to 0.12)	0.09 (−0.03 to 0.20)
Northern Ireland	0.05 (−0.03 to 0.12)	−0.15 (−0.84 to 0.55)	**−0.19 (−0.34 to −0.04)**	0.23 (−0.29 to 0.76)	**0.15 (0.02−0.28)**

Data are presented as regression coefficients (95% CI); values shown in bold indicate statistically significant results (*P* < 0.05).

### Monthly DIDs

**Fig. 2:**
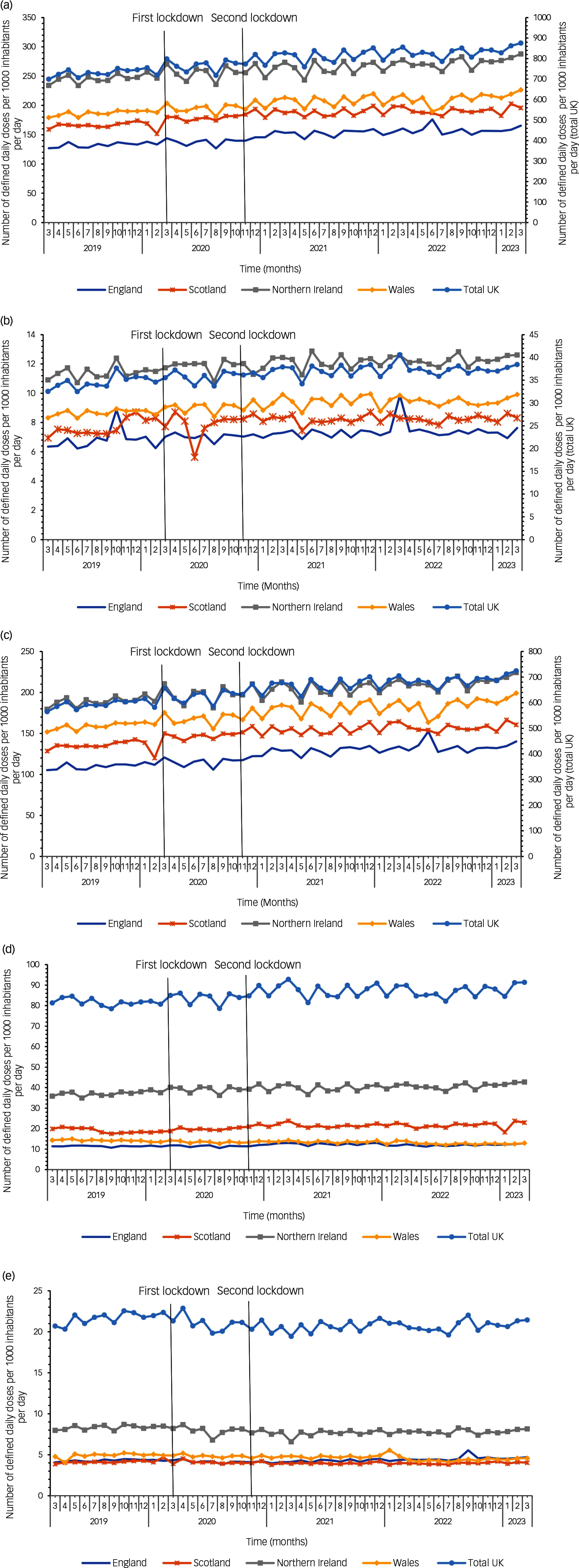
Prescribing pattern of psychotropic medications expressed as the daily dispensed doses per 1000 inhabitants per day (DID) for the UK and each UK country from March 2019 to March 2023. (a) All psychotropic medications, (b) antipsychotics, (c) antidepressants, (d) anxiolytics/hypnotics and (e) antidementia drugs.

Patterns observed with dose-standardised measures largely mirrored NTI trends. Antidepressant utilisation increased across all UK nations, with Northern Ireland consistently showing the highest levels (Table 3; Fig. 2). Relative increases were most pronounced in England and Scotland. Anxiolytic and hypnotic utilisation increased in Scotland and Northern Ireland, but declined in England and Wales. Overall changes were modest and clinically small. During the early lockdown period, DIDs increased despite reductions in prescription counts, indicating that fewer prescriptions were issued, but at higher doses or longer durations. Antipsychotic utilisation increased significantly in all countries except Wales. Northern Ireland again showed the highest utilisation levels, with values nearly double those observed in Scotland. Baseline trends were significant for both the UK (*β*_1_ = 0.24; 95% CI 0.03–0.44) and Scotland (*β*_1_ = 0.12; 95% CI 0.04–0.20). Antidementia medicine utilisation increased modestly overall (3.6% across the UK). Scotland, England and Northern Ireland showed significant increases, whereas Wales demonstrated a 4.3% decrease. Segmented regression indicated an immediate increase following the first lockdown (*β*_2_ = 0.33; 95% CI 0.04–0.61), followed by sustained upward trends after both lockdowns in most countries.

**Table 3:** Segmented regression analysis of the monthly defined daily doses per 1000 inhabitants per day for psychotropic medications across England, Scotland, Wales and Northern Ireland over the study period March 2019 to March 2023 Table 3 long description.

Psychotropic medications	Baseline trend (*β*_1_)	Level change immediately after first lockdown (*β*_2_)	Time trend after first lockdown (*β*_3_)	Level change immediately after second lockdown (*β*_4_)	Time trend after second lockdown (*β*_5_)
All					
UK	2.60 (−1.50 to 6.60)	24.40 (−21.50 to 70.30)	−3.10 (−11.50 to 5.40)	27.50 (−8.70 to 63.70)	2.40 (−5.20 to 9.90)
England	0.70 (−0.30 to 1.60)	2.60 (−8.20 to 13.50)	−0.90 (−2.90 to 1.10)	**1.80 (0.01−2.30)**	0.70 (−1.10 to 2.50)
Scotland	0.05 (−0.90 to 1.03)	11.10 (−0.01 to 22.20)	0.30 (−1.80 to 2.30)	6.40 (−2.30 to 15.20)	−0.10 (−1.90 to 1.70)
Wales	0.80 (−0.50 to 2.10)	4.00 (−10.70 to 18.60)	−0.80 (−3.60 to 1.90)	6.60 (−4.90 to 18.20)	0.50 (−1.90 to 2.90)
Northern Ireland	1.00 (−0.50 to 2.60)	6.70 (−10.90 to 24.40)	−1.60 (−4.80 to 1.70)	3.60 (−10.30 to 17.60)	1.30 (−1.60 to 4.20)
Antipsychotics					
UK	**0.24 (0.03−0.44)**	−0.22 (−2.57 to 2.13)	−0.22 (−0.65 to 0.22)	0.89 (−0.97 to 2.74)	0.02 (−0.36 to 0.41)
England	0.05 (−0.04 to 0.14)	0.009 (−1.04 to 1.06)	−0.06 (−0.26 to 0.13)	0.23 (−0.61 to 1.06)	0.02 (−0.15 to 0.2)
Scotland	**0.12 (0.04−0.2)**	−0.71 (−1.6 to 0.19)	−0.09 (−0.26 to 0.07)	0.31 (−0.40 to 1.01)	−0.03 (−0.17 to 0.12)
Wales	0.02 (−0.03 to 0.08)	0.20 (−0.42 to 0.82)	−0.02 (−0.14 to 0.09)	0.27 (−0.23 to 0.76)	0.005 (−0.1 to 0.11)
Northern Ireland	0.04 (−0.03 to 0.11)	0.28 (−0.52 to 1.08)	−0.04 (−0.19 to 0.11)	0.09 (−0.54 to 0.72)	0.02 (−0.11 to 0.15)
Antidepressants					
UK	1.59 (−2.39 to 5.57)	14.88 (−15.63 to 45.39)	1.11 (−3.19 to 5.40)	1.59 (−26.21 to 29.38)	−1.16 (−3.78 to 1.47)
England	0.52 (−0.49 to 1.53)	−1.65 (−9.39 to 6.09)	0.49 (−0.60 to 1.58)	0.01 (−7.04 to 7.06)	**−0.82 (−1.49 to −0.16)**
Scotland	−0.17 (−1.15 to 0.81)	**11.30 (3.77−18.84)**	0.64 (−0.42 to 1.70)	2.38 (−4.48 to 9.24)	−0.45 (−1.10 to 0.20)
Wales	0.72 (−0.64 to 2.08)	1.84 (−8.61 to 12.30)	0.09 (−1.38 to 1.57)	−2.14 (−11.66 to 7.39)	−0.16 (−1.06 to 0.74)
Northern Ireland	0.53 (−0.92 to 1.98)	3.38 (−7.74 to 14.49)	−0.12 (−1.68 to 1.45)	1.33 (−8.79 to 11.46)	0.28 (−0.68 to 1.23)
Anxiolytics/hypnotics					
UK	−0.20 (−0.50 to 0.30)	3.80 (−1.50 to 9.20)	−0.01 (−0.90 to 1.00)	3.80 (−0.40 to 8.00)	0.20 (−0.70 to 1.10)
England	−0.01 (−0.10 to 0.07)	0.40 (−0.60 to 1.30)	−0.04 (−0.20 to 0.10)	**1.00 (0.30−1.80)**	0.05 (−0.10 to 0.20)
Scotland	−0.20 (−0.40 to 0.06)	1.50 (−0.40 to 3.50)	0.40 (−0.01 to 0.70)	1.30 (−0.20 to 2.90)	−0.10 (−0.40 to 0.20)
Wales	**−0.09 (−0.20 to −0.01)**	0.40 (−0.50 to 1.30)	−0.03 (−0.20 to 0.10)	**0.90 (0.02−2.00)**	0.08 (−0.07 to 0.30)
Northern Ireland	0.20 (−0.01 to 0.40)	1.60 (−1.10 to 4.20)	−0.30 (−0.70 to 0.20)	0.60 (−1.50 to 2.70)	0.20 (−0.20 to 0.60)
Antidementia					
UK	0.13 (−0.02 to 0.27)	0.58 (−0.75 to 1.92)	**−0.53 (−0.82 to −0.25)**	0.52 (−0.48 to 1.53)	**0.42 (0.17−0.66)**
England	0.03 (−0.02 to 0.07)	0.03 (−0.37 to 0.44)	**−0.10 (−0.18 to −0.01)**	0.04 (−0.26 to 0.34)	**0.09 (0.02−0.16)**
Scotland	0.02 (−0.01 to 0.05)	**0.33 (0.04−0.61)**	**−0.10 (−0.17 to −0.04)**	0.04 (−0.18 to 0.25)	**0.09 (0.03−0.14)**
Wales	−0.01 (−0.07 to 0.06)	−0.11 (−0.74 to 0.51)	−0.03 (−0.16 to 0.10)	−0.35 (−0.82 to 0.12)	0.09 (−0.03 to 0.20)
Northern Ireland	0.02 (−0.06 to 0.10)	0.35 (−0.37 to 1.08)	**−0.22 (−0.38 to −0.07)**	0.28 (−0.27 to 0.82)	**0.21 (0.07−0.34)**

Data are presented as regression coefficients (95% CI); values shown in bold indicate statistically significant results (*P* < 0.05).

## Discussion

### Principal findings

This study provides a national comparative analysis of psychotropic prescribing across the four UK nations before and during the COVID-19 pandemic. Overall utilisation of antidepressants, anxiolytics and hypnotics, antipsychotics and antidementia medicines increased between 2019 and 2023. However, interrupted time-series analysis indicated that most changes occurred gradually rather than as abrupt shifts associated with national lockdowns. These findings suggest that prescribing patterns during the pandemic largely reflected continuation or amplification of existing trends, rather than immediate behavioural responses to lockdown measures. Antidepressants showed the largest increase in utilisation across all UK nations. This pattern is consistent with well-documented increases in psychological distress during the pandemic, including higher prevalence of anxiety and depressive symptoms in population surveys.^6–8^ However, these increases also occurred against a background of rising antidepressant prescribing before the pandemic, with annual growth of approximately 5% reported in Scotland during the preceding decade.^27^ The observed increases therefore likely reflect both pre-existing prescribing trajectories and additional pressures on mental health services during the pandemic.

### Findings in context

Psychotropic prescribing in the UK is strongly shaped by primary care practice, where most antidepressants and anxiolytic or hypnotic medicines are initiated and managed. Consequently, changes in prescribing patterns may reflect shifts in help-seeking behaviour, accessibility of psychological therapies and service disruption during the pandemic. Similar increases in antidepressant utilisation have been reported in other European countries during the COVID-19 period.^11–13,28^

Trends in anxiolytic and hypnotic prescribing were more heterogeneous. Scotland and Northern Ireland showed modest increases, whereas Wales demonstrated reductions over the study period. These differences may reflect national prescribing initiatives aimed at reducing benzodiazepine and non-benzodiazepine drug use.^16^ In Scotland, apparent increases should also be interpreted in the context of long-term reductions in traditional hypnotics alongside increasing use of melatonin, suggesting possible substitution within the therapeutic class.^28^ Antipsychotic prescribing increased gradually during the study period, particularly in Northern Ireland. Importantly, this increase is unlikely to reflect rising incidence of psychotic disorders. Evidence suggests that antipsychotics are increasingly prescribed in general practice for non-psychotic indications, including behavioural symptoms associated with dementia and other neuropsychiatric conditions.^29^ The pandemic may have further influenced prescribing through reduced access to specialist review, increased clinical uncertainty and challenges in monitoring patients with complex mental health needs. Antidementia prescribing increased modestly overall, but varied across the UK nations. Early reductions following the first lockdown are consistent with disruption to diagnostic pathways and reduced access to specialist services during the pandemic.^14,30^ Subsequent increases likely reflect recovery of dementia assessment and treatment services, rather than a direct causal effect of COVID-19 infection on dementia incidence.

### Cross-national differences within the UK

Substantial differences in prescribing patterns were observed between UK nations. Northern Ireland consistently demonstrated higher utilisation across most psychotropic medicine classes. These differences may reflect variation in population characteristics, underlying prevalence of mental health conditions or differences in service organisation across devolved health systems.^18^ Wales showed several counter-directional trends, including reductions in anxiolytic and antidementia prescribing, which may reflect national prescribing policies and optimisation initiatives. Differences in underlying mental health burden across populations may also have contributed to the observed prescribing variation. Population surveys conducted during the pandemic identified substantial increases in anxiety and depressive symptoms across the UK, particularly among younger adults, women and socioeconomically disadvantaged groups.^6,7^ International evidence similarly demonstrated marked increases in common mental disorders during the COVID-19 period, with the World Health Organization estimating a 25% global increase in the prevalence of anxiety and depression during the first year of the pandemic.^8^ A recent systematic review further highlighted persistent increases in psychological distress across multiple countries following COVID-19-related social and healthcare disruption.^9^ These wider epidemiological trends may partly explain the sustained increases in psychotropic utilisation observed across several UK nations during the study period.

### Strengths and limitations

A major strength of this study is the use of comprehensive national data-sets capturing all community-dispensed psychotropic medicines across the four UK nations. The analysis covers a 4-year period spanning the pre-pandemic and pandemic phases, allowing examination of both immediate and longer-term changes in prescribing patterns. The use of two complementary utilisation metrics (monthly NTIs and DIDs) enabled more nuanced interpretation of prescribing behaviour, accounting for both dispensing frequency and treatment intensity. However, several limitations should be considered. The PCA data-sets provide aggregated dispensing data and do not include information on clinical indications, treatment duration or patient-level characteristics such as age, gender or comorbidities. Consequently, the analysis cannot determine whether prescribing changes reflect specific psychiatric diagnoses or other clinical indications. The data also do not allow differentiation between newly initiated and continuing treatment. Aggregated analyses may therefore mask important patient-level dynamics. Nevertheless, similar population-level data-sets have been widely used to evaluate prescribing trends and the effects of health system changes during the COVID-19 pandemic.^31–34^ Future research should incorporate linked, patient-level data-sets to examine prescribing trends according to specific psychiatric diagnoses, demographic characteristics and socioeconomic status, as well as to distinguish treatment initiation from continuation. Such analyses would help identify population groups most affected by pandemic-related disruption, assess potential inequalities in access to mental healthcare and evaluate the appropriateness and longer-term outcomes of psychotropic prescribing during periods of health system stress.

### Clinical implications

The sustained increase in psychotropic medication use observed during and following the pandemic period up to March 2023 highlights substantial mental health needs within the population and pressures on mental health services during this extended time frame. The absence of abrupt prescribing changes following lockdowns suggests that long-term structural pressures on mental healthcare, rather than short-term restrictions, were the primary drivers of observed trends. Strengthening the resilience of mental health services is therefore essential. The findings also provide an important benchmark for evaluating longer-term post-pandemic psychotropic prescribing trends and future responses to large-scale healthcare disruption. Continued surveillance using more recent post-2023 prescribing data will be important to determine whether the trends identified in this study persisted, stabilised or reversed following longer-term recovery of healthcare services and mental healthcare pathways. This is particularly relevant, given emerging evidence from Scotland showing the reversal and recovery of pandemic-related disruptions in prescribing and healthcare utilisation for other long-term conditions, including hypertension and hypercholesterolaemia, during the later post-pandemic period.^35^ Improving access to psychological therapies, supporting continuity of care for people with severe mental illness and maintaining diagnostic and treatment pathways for dementia during periods of service disruption should be priorities. Future research using linked, patient-level data-sets is needed to examine prescribing appropriateness, distinguish treatment initiation from continuation and identify population groups most affected by pandemic-related changes in mental healthcare.

In conclusion, this national analysis of community prescribing across the UK shows that utilisation of psychotropic medicines increased between 2019 and 2023, with antidepressants demonstrating the largest rise. Interrupted time-series analysis indicated that most changes occurred gradually and were not strongly associated with the timing of national COVID-19 lockdowns, suggesting that prescribing patterns largely reflected continuation of existing trends rather than immediate responses to public health restrictions. Marked differences between UK nations highlight the influence of local prescribing policies, service organisation and population characteristics. The findings emphasise the sustained demand for mental healthcare during and after the pandemic period up to March 2023, and underline the importance of resilient mental health and dementia services capable of maintaining continuity of care during periods of health system disruption. Further research using linked patient-level data is needed to understand prescribing appropriateness and longer-term clinical outcomes.

## Supporting information

10.1192/bjo.2026.12054.sm001 Kurdi et al. supplementary material Kurdi et al. supplementary material

## Data Availability

The data used in this study are publicly available, aggregated prescribing data-sets obtained from national PCA sources for England, Scotland, Wales and Northern Ireland. These data-sets are available from the respective National Health Service (NHS) data portals: NHS Business Services Authority (England), Public Health Scotland Open Data, NHS Wales Shared Services Partnership and the Business Services Organisation Northern Ireland. The data analysed during the current study are publicly accessible and can be obtained from the relevant data providers through the links provided in the reference list. No individual-level or identifiable patient data were used. The statistical code used for the interrupted time-series analysis can be made available from the corresponding author, A.K., upon reasonable request to facilitate replication of the analysis.

## Supplementary material

The supplementary material is available online at https://doi.org/10.1192/bjo.2026.12054

## Fig. 1 Long description

The image contains five line graphs showing the prescribing patterns of different psychotropic medications in the UK and its constituent countries from March 2019 to March 2023. Each graph represents a different category of medication: all psychotropic medications, antipsychotics, antidepressants, anxiolytics/hypnotics, and antidementia drugs. The x-axis for all graphs represents time in months, while the y-axis represents the number of items dispensed per 1000 inhabitants. Each graph includes lines for England, Scotland, Northern Ireland, Wales, and the total UK. The first and second lockdown periods are marked with vertical lines. Panel A: This graph shows the total number of items dispensed for all psychotropic medications. The lines for each country show fluctuations over time, with noticeable changes around the lockdown periods. Panel B: This graph depicts the dispensing patterns for antipsychotics. Similar to Panel A, there are fluctuations, with some variations between the countries. Panel C: This graph shows the dispensing patterns for antidepressants. The trends are relatively stable with slight increases over time. Panel D: This graph illustrates the dispensing patterns for anxiolytics/hypnotics. The lines show some variability, with noticeable changes around the lockdown periods. Panel E: This graph represents the dispensing patterns for antidementia drugs. The trends are relatively stable with minor fluctuations. Overall, the graphs indicate changes in medication dispensing patterns during the COVID-19 pandemic, particularly around the lockdown periods.

Navigate back to Fig. 1.

## Table 1 Long description

A table comparing the utilization trends of psychotropic medications across England, Scotland, Wales, and Northern Ireland from March 2019 to March 2023. The table has 16 rows and 5 columns. Column headers are UK (total), England, Scotland, Wales, and Northern Ireland. Row labels include categories for different types of psychotropic medications and their changes in utilization. Row 1: Number of dispensed items per 1000 inhabitants. Row 2: All psychotropic medications, Absolute change (relative % change), 193.0 (24.6%), 38.2 (26.0%), 37.3 (22.0%), 48.9 (23.3%), 53.2 (19.5%). Row 3: All psychotropic medications, Monthly average change, 2.8*, 0.7*, 0.6*, 0.7*, 0.7*. Row 4: Antipsychotics, Absolute change (relative % change), 27.9 (50.4%), 2.5 (16.5%), 3.2 (26.6%), 3.4 (16.6%), 3.4 (15.0%). Row 5: Antipsychotics, Monthly average change, 0.2*, 0.01*, 0.03*, 0.04*, 0.04*. Row 6: Antidepressants, Absolute change (relative % change), 123.9 (24.6%), 32.2 (31.2%), 18.8 (20.0%), 37.7 (23.3%), 35.1 (24.2%). Row 7: Antidepressants, Monthly average change, 2.6*, 0.7*, 0.6*, 0.7*, 0.6*. Row 8: Anxiolytics/hypnotics, Absolute change (relative % change), 3.8 (2.7%), 0.5 (2.5%), 1.9 (7.3%), -3.1 (-9.5%), 4.6 (7.4%). Row 9: Anxiolytics/hypnotics, Monthly average change, -0.1*, -0.01*, 0.02*, -0.1*, 0.01*. Row 10: Antidementia, Absolute change (relative % change), 2.0 (9.3%), 0.01 (1.7%), 0.3 (7.5%), 1.4 (29.0%), 0.3 (3.7%). Row 11: Antidementia, Monthly average change, 0.02*, 0.01*, -0.001*, 0.02*, -0.01*. Row 12: Number of defined daily doses per 1000 inhabitants per day. Row 13: All psychotropic medications, Absolute change (relative % change), 175.9 (25.1%), 38.4 (30.3%), 36.6 (23.0%), 47.2 (26.3%), 53.7 (23.0%). Row 14: All psychotropic medications, Monthly average change, 2.9*, 0.7*, 0.7*, 0.7*, 0.8*. Row 15: Antipsychotics, Absolute change (relative % change), 5.9 (18.2%), 1.3 (20.0%), 1.4 (19.7%), 1.6 (19.1%), 1.7 (15.6%). Row 16: Antipsychotics, Monthly average change, 0.08*, 0.02*, 0.02*, 0.02*, 0.02*. Row 17: Antidepressants, Absolute change (relative % change), 159.1 (28.2%), 35.0 (33.3%), 32.0 (24.9%), 47.3 (31.1%), 44.9 (25.0%). Row 18: Antidepressants, Monthly average change, 2.6*, 0.7*, 0.6*, 0.7*, 0.6*. Row 19: Anxiolytics/hypnotics, Absolute change (relative % change), 10.0 (12.3%), 1.5 (13.3%), 3.0 (15.3%), -1.4 (-10.0%), 6.9 (19.3%). Row 20: Anxiolytics/hypnotics, Monthly average change, 0.2*, 0.02*, 0.07*, -0.04*, 0.1*. Row 21: Antidementia, Absolute change (relative % change), 0.7 (3.6%), 0.6 (15.1%), 0.2 (4.2%), -0.2 (-4.3%), 0.2 (2.1%). Row 22: Antidementia, Monthly average change, -0.02*, 0.01*, -0.003*, -0.01*, -0.01*.

Navigate back to Table 1.

## Table 3 Long description

The table presents data on psychotropic medication usage trends across the UK regions during lockdown periods. It includes baseline trends, level changes immediately after the first and second lockdowns, and time trends after each lockdown for various medications. The table has 5 columns: Baseline trend (β_1_), Level change immediately after first lockdown (β_2_), Time trend after first lockdown (β_3_), Level change immediately after second lockdown (β_4_), and Time trend after second lockdown (β_5_). The rows are categorized by medication type and region. Row 1: All, UK: 2.60, 24.40, -3.10, 27.50, 2.40. Row 1: All, England: 0.70, 2.60, -0.90, 1.80, 0.70. Row 1: All, Scotland: 0.05, 11.10, 0.30, 6.40, -0.10. Row 1: All, Wales: 0.80, 4.00, -0.80, 6.60, 0.50. Row 1: All, Northern Ireland: 1.00, 6.70, -1.60, 3.60, 1.30. Row 2: Antipsychotics, UK: 0.24, -0.22, -0.22, 0.89, 0.02. Row 2: Antipsychotics, England: 0.05, 0.09, -0.06, 0.23, 0.02. Row 2: Antipsychotics, Scotland: 0.12, -0.71, -0.09, 0.31, -0.03. Row 2: Antipsychotics, Wales: 0.07, 0.20, -0.02, 0.27, 0.005. Row 2: Antipsychotics, Northern Ireland: 0.04, 0.28, -0.04, 0.09, 0.02. Row 3: Antidepressants, UK: 1.59, 14.88, 1.11, 1.59, -1.16. Row 3: Antidepressants, England: 0.52, -1.65, 0.49, 0.01, -0.82. Row 3: Antidepressants, Scotland: -0.17, 11.30, 0.64, 2.38, -0.45. Row 3: Antidepressants, Wales: 0.72, 1.84, 0.97, -2.14, -0.16. Row 3: Antidepressants, Northern Ireland: 0.53, 3.38, -0.12, 1.33, 0.28. Row 4: Anxiolytics/hypnotics, UK: -0.20, 3.80, -0.01, 3.80, 0.20. Row 4: Anxiolytics/hypnotics, England: -0.01, 0.40, -0.04, 1.00, 0.05. Row 4: Anxiolytics/hypnotics, Scotland: -0.20, 1.50, 0.40, 1.30, 0.10. Row 4: Anxiolytics/hypnotics, Wales: -0.09, 0.40, -0.03, 0.90, 0.08. Row 4: Anxiolytics/hypnotics, Northern Ireland: 0.20, 1.60, -0.30, 0.60, 0.20. Row 5: Antidementia, UK: 0.13, 0.58, -0.53, 0.52, 0.42. Row 5: Antidementia, England: 0.03, 0.03, -0.10, 0.04, 0.09. Row 5: Antidementia, Scotland: 0.02, 0.33, -0.10, 0.04, 0.09. Row 5: Antidementia, Wales: -0.01, -0.11, -0.03, -0.35, 0.09. Row 5: Antidementia, Northern Ireland: 0.02, 0.35, -0.22, 0.28, 0.21.

Navigate back to Table 3.
