# Supplementary material for: Psychotropic prescribing in UK primary care during the COVID-19 pandemic: national interrupted time-series analysis
Source: BJPsych Open. 2026 Jul 28;12(4):e201. doi: 10.1192/bjo.2026.12054 (PMC13419623; doi:10.1192/bjo.2026.12054)
Supplement: Kurdi et al. supplementary material [file S2056472426120547sup001.docx]

**Supplementary File 1. List of all psychotropic medications included in the study**

| **Drug Class** | **Specific drugs within this class** |
| --- | --- |
| Anxiolytics/Hypnotics | Benzodiazepines:  Alprazolam  Chlordiazepoxide  Diazepam  Lormetazepam  Loprazolam  Nitrazepam  Oxazepam  Temazepam  Non-benzodiazepines:  Buspirone  Chloral hydrate  Clomethiazole  Melatonin  Meprobamate  Zolpidem  Zopiclone |
| Antipsychotics | Amisulpride  Aripiprazole Benperidol  Cariprazine  Chlorpromazine hydrochloride  Chlorprothixene  Clozapine  Flupentixol hydrochloride  Fluphenazine hydrochloride  Haloperidol  Levomepromazine hydrochloride  Levomepromazine maleate  Loxapine succinate  Lurasidone  Melperone hydrochloride  Olanzapine  Paliperidone  Pericyazine  Perphenazine  Pimozide  Promazine hydrochloride  Quetiapine  Risperidone  Sulpiride  Thioridazine  Trifluoperazine  Ziprasidone hydrochloride  Zotepine  Zuclopenthixol acetate  Zuclopenthixol hydrochloride |
| Antidepressants | TCA:  Amitriptyline hydrochloride  Clomipramine hydrocholoride  Dosulepin hydrochloride  Doxepin  Imipramine hydrochloride  Lofepramine hydrochloride  Mianserin hydrochloride  Nortriptyline  Trimipramine maleate  MOAI:  Isocarboxazid  Moclobemide  Phenelzine sulphate  Tranylcypromine sulphate  SSRI:  Citalopram hydrobromide  Citalopram hydrochloride  Escitalopram  Fluoxetine hydrochloride  Fluvoxamine maleate  Paroxetine hydrochloride  Sertraline hydrochloride  Other:  Duloxetine hydrochloride  Venlafaxine  Agomelatine  Mirtazapine  Vortioxetine  Trazodone hydrochloride |
| Drugs for dementia | AChEI:  Donepezil  Galantamine  Rivastigmine  NMDAR antagonists:  Memantine |
